# Childhood ADHD and subthreshold symptoms are associated with cognitive functioning at age 40—a cohort study on perinatal birth risks

**DOI:** 10.3389/fpsyg.2024.1393642

**Published:** 2024-08-29

**Authors:** Nella Schiavone, Maarit Virta, Sami Leppämäki, Jyrki Launes, Ritva Vanninen, Annamari Tuulio-Henriksson, Ilkka Järvinen, Eliisa Lehto, Laura Hokkanen

**Affiliations:** ^1^Department of Psychology and Logopedics, University of Helsinki, Helsinki, Finland; ^2^Terveystalo Healthcare, Helsinki, Finland; ^3^Department of Clinical Radiology, Kuopio University hospital, Kuopio, Finland; ^4^Clinical Radiology, School of Medicine, University of Eastern Finland, Kuopio, Finland

**Keywords:** ADHD, cohort study, cognition, neuropsychological assessment, deficit, adult

## Abstract

**Introduction:**

In this prospective cohort study over 40 years we investigated the effect of childhood attention-deficit/hyperactivity disorder (ADHD) and subthreshold ADHD on cognitive performance in adulthood.

**Methods:**

The cohort comprised individuals with mild perinatal risks. Childhood ADHD group (cADHD, *n* = 39) was compared to a group with subthreshold childhood attention or hyperactivity symptoms (cAP; *n* = 79), a group with similar perinatal risks but no ADHD symptoms (*n* = 255), and to controls without ADHD symptoms or perinatal risks (*n* = 69). The groups were assessed with multiple neuropsychological measures in domains of verbal reasoning, perceptual skills, memory, working memory, attention, executive functions, and speed. Group-level differences and frequencies of deficient functioning were analyzed.

**Results:**

Overall, the groups’ performance differed in all cognitive domains at age 40. Verbal reasoning, perceptual skills, memory, and speed had the largest effect sizes (0.51–0.62). The cADHD group’s performance was lower than the other groups’ on 13 out of 21 measures. The cAP group performed poorer than controls on five measures. In the cADHD group, 23% had three or more deficient cognitive domains, compared to 4–6% in the other groups.

**Discussion:**

Childhood ADHD is associated with impaired cognitive functioning in adulthood on several cognitive domains whereas childhood subthreshold ADHD is linked to fewer cognitive deficits. Task complexity was linked to poorer performance within the ADHD group. Our results add to the scarce longitudinal evidence of cognitive outcomes related to childhood ADHD and subthreshold symptoms.

## 1 Introduction

Attention-deficit/hyperactivity disorder (ADHD) has a prevalence of 3–5% in children and affects individuals negatively throughout the lifespan (Barkley et al., 2006; Biederman et al., 2012; Klein et al., 2012; Moffitt et al., 2015; Fayyad et al., 2017). We have previously shown associations between childhood ADHD and poor academic outcomes and higher mortality in our study cohort (Schiavone et al., 2019, 2022). Poor performance in various cognitive domains in adult ADHD has been reported in several cross-sectional and longitudinal studies (e.g., Biederman et al., 2009; Fuermaier et al., 2015). Meta-analytic reviews have found impairments in intelligence (Pievsky and McGrath, 2018), focused and sustained attention (Schoechlin and Engel, 2005; Bálint et al., 2008; Mowinckel et al., 2015), working memory (Alderson et al., 2013), and verbal memory (Schoechlin and Engel, 2005; Skodzik et al., 2017) to have the largest effect sizes when comparing adults with ADHD to controls. Problems in executive functions (EF) have been proposed to be central in ADHD (Barkley, 1997) and there is evidence for weaker EF performance in verbal fluency, set shifting and inhibition (Boonstra et al., 2005, 2010; Pievsky and McGrath, 2018). In addition to weaknesses in cognitive domains requiring complex cognitive skills, adults with ADHD have slower processing speed (Nigg et al., 2005a; Anker et al., 2022) and have more variability in reaction time (Kofler et al., 2013; Pievsky and McGrath, 2018). Despite group-level differences on several cognitive tasks between ADHD adults and controls, heterogeneity in performance across cognitive domains is a core characteristic of ADHD (Nigg et al., 2005b; Mostert et al., 2015; Leib et al., 2021).

The difference in cognitive performance between subjects with ADHD and controls may change over time. One meta-analysis suggests that cognitive difficulties remain similar in children, adolescents, and adults with ADHD (Frazier et al., 2004). According to the few longitudinal studies available, cognitive performance remains lower over time both from childhood to adulthood and within adulthood (Fischer et al., 2005; Barkley et al., 2008; Biederman et al., 2009, 2012; Miller et al., 2012; Moffitt et al., 2015). Another review of meta-analyses, however, suggests cognitive differences between subjects with ADHD and controls to diminish in adolescence but to increase again after young adulthood (Pievsky and McGrath, 2018). Only 15% of the studies in the review included subjects over the age of 14 (Pievsky and McGrath, 2018), which highlights the need for more information on adult cognitive functioning. Furthermore, studies evaluating cognitive performance and impairment in adults aged 40 or older with known childhood ADHD are still scarce (Seidman, 2006; Franke et al., 2018).

One approach in estimating cognitive functioning is to study cognitive impairment. This can be achieved by identifying the proportion of individuals that fall below a cutoff point determined by control or normative group performance (Nigg et al., 2005b; Coghill et al., 2014). With this approach, children, adolescents, and adults with ADHD have exhibited more cognitive deficits than controls (Biederman et al., 2004; Fuermaier et al., 2015; Mostert et al., 2015; Halleland et al., 2019). In two studies, executive dysfunction was present in 24 to 31% of adults with ADHD (Biederman et al., 2006; Halleland et al., 2019). In another study, 89% of ADHD adults had at least a mild impairment in executive function and attention measures (Mostert et al., 2015). Identifying cognitive deficits has implications for designing and targeting support and interventions. Studies focusing on cognitive deficits to examine impairment in adults with childhood ADHD are lacking and have mainly focused on executive functions and attention.

Recently, ADHD symptoms below the diagnostic threshold and their adverse effect have gained more research focus (Matte et al., 2012; Balázs and Keresztény, 2014; Kirova et al., 2019). In children and adolescents, such subthreshold symptoms have been linked to similar impairments in functioning (e.g., comorbid psychiatric disorders, educational adversity) and cognition as in the full disorder (Balázs and Keresztény, 2014; Kirova et al., 2019). One study in children suggested that basic information processing skills are linearly associated with ADHD symptom severity (Salum et al., 2014). Studies of adult cognitive performance associated with subthreshold levels of ADHD symptoms are rare. These studies have found no difference between adults with subthreshold symptoms and controls in executive functions (Faraone et al., 2006; Schneidt et al., 2020). In one study, a subthreshold ADHD group performed worse than controls on verbal IQ (Faraone et al., 2006). More information is needed about cognitive deficits in adulthood associated with childhood subthreshold ADHD symptoms.

ADHD is a highly heritable disorder, but it is also associated with environmental factors, especially related to pregnancy, birth, and childhood (Thapar et al., 2012; Kooij et al., 2019). A population-based study indicated that pre- and perinatal risks, especially preterm birth, low birth weight, and low Apgar scores, increase the risk of ADHD in adulthood (Halmøy et al., 2012). These adverse events before and during birth engender negative impact on cognitive development and functioning. For example, adults born prematurely or with a low birth weight perform more poorly than controls on cognitive tests (Allin et al., 2011; Pyhälä et al., 2011), and babies with low Apgar score tend to have lower cognitive functioning in young adulthood (Ehrenstein et al., 2009). Little is yet known about the impact of perinatal risks on cognition in adulthood in individuals with childhood ADHD.

Our aim was to provide a comprehensive investigation of the cognitive performance of 40-year-old adults with childhood ADHD or subthreshold symptoms and perinatal risks by examining various cognitive domains and cognitive deficits. We compared study subjects to cohort members with similar birth risks and to controls. Based on previous studies, we hypothesized the ADHD group to perform worse on cognitive measures and to exhibit more deficits than controls, and the subthreshold symptom group to fall in the middle of the continuum of cognitive performance between controls and the childhood ADHD.

## 2 Materials and methods

### 2.1 Participants

Four groups were compared in this study: childhood ADHD group (cADHD; *n* = 39), childhood attention problems group, (cAP; *n* = 79), cohort group without childhood attention problems, (Non-cAP; *n* = 255), and control group (*n* = 69). The study participants are part of a cohort prospectively followed from birth. All perinatal risk cohort members (*N* = 1,196) were born in one maternity hospital in Helsinki between 1971 and 1974. Participants were included in the longitudinal study if they had one or more predefined perinatal risks including hyperbilirubinemia, birth weight below 2000 grams, Apgar score < 7, respiratory distress requiring external ventilation, maternal diabetes, hypoglycemia, septicemia, or neurological symptoms such as rigidity, apnea, hyperexitability, convulsions or prolonged feeding difficulty in the absence of other risks. The birth risks were considered mild and typically did not cause major disability. Participants with disability, e.g., cerebral palsy, blindness, or malformations, were excluded from follow-up. A control group with no perinatal risks born in the same maternity hospital has also been followed from childhood. The complete study protocol, attrition, and definitions of birth risks are described elsewhere (Hokkanen et al., 2013; Launes et al., 2014). The project was approved by Ethical Review Board of the Helsinki and Uusimaa hospital district (number 147/13/3/00/2013) and written informed consent was gathered from all participants.

**Figure 1:**
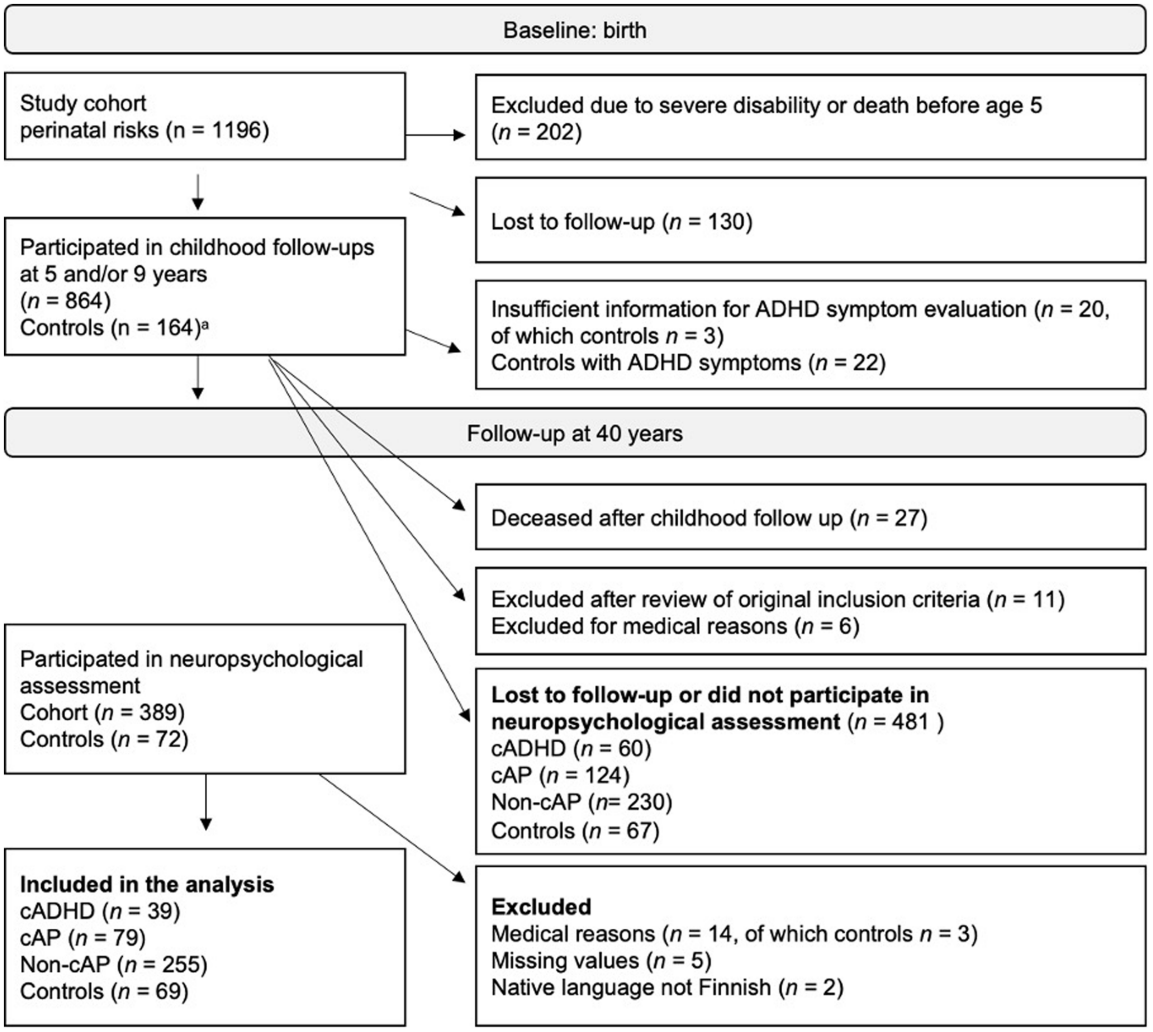
Participation from birth to the 40-year follow-up. cADHD, childhood ADHD; cAP, childhood attention problem; Non-cAP, no childhood ADHD or attention problems. ^a^The control group was recruited during the 5-year follow-up.

For the latest follow-up, participants with known contact details were contacted by mail. Participant flow from birth is illustrated in Figure 1. A total of 442 subjects were analyzed in the present study. Participants were excluded if they had medical reasons possibly causing neuropsychological impairment (traumatic brain injury, schizophrenia, HIV). Brain MRI was used to exclude participants due to traumatic brain injuries or strokes. Visual assessment of the images was performed by a specialist in neuroradiology (RV). Subjects with 19% or more missing tests or two out of three missing outcome measures within the same cognitive domain were excluded from the analysis.

### 2.2 Definition of childhood ADHD and attention problems

Childhood ADHD was determined retrospectively according to DSM-IV criteria by a pediatrician (the principal investigator at the time, Katarina Michelsson) as ADHD was not a diagnosis used in Finland during the childhood follow-ups (American Psychiatric Association, 2000; Tervo et al., 2017). The participants were diagnosed using all available childhood data, including behavioral observations by a doctor, speech therapist, and psychologist during assessment situations, and questionnaires filled in by parents and day care and school personnel (Hokkanen et al., 2013). Subthreshold ADHD symptoms in childhood were similarly evaluated during the latest follow-up. All childhood information described above was used to classify individuals who did not have ADHD in childhood into two groups: no or low levels of attention problems (Non-cAP) and attention problems (cAP). The group with attention problems represents individuals with subthreshold ADHD symptoms encompassing attention and/or hyperactivity. An individual was classified as having attention problems in childhood, if they presented clear symptoms in at least one environment or some symptoms in two environments (home, day care/ school, or research appointments). None of the participants were medicated or had history of medication for ADHD. The exact protocol for classifying individuals is described elsewhere (Schiavone et al., 2019).

### 2.3 Measures

The participants filled in a questionnaire about current life situation online or on paper. Number of birth risks were stratified into three classes: one, two, or three to five birth risks. Childhood socioeconomic status (SES) was calculated as the median highest status of either mother or father with class 1 representing the highest level (Statistical Office of the City of Helsinki, 1954, 1975). Four SES classes were originally used, but two of the lowest classes were combined due to low expected cell counts in contingency table analyses. Education was stratified into three classes where class 1 represents basic education (up to 9 years), class 2 secondary education (up to 12 years) and class 3 tertiary education (13+ years). There were two missing values in education, which were imputed with the median value of the childhood group. Current ADHD symptoms were measured with the World Health Organization Adult ADHD Self-Report Screening Scale, ASRS- v1.1 (Kessler et al., 2005). The ASRS includes six questions estimating ADHD symptoms of inattention and hyperactivity following DSM-IV criteria (American Psychiatric Association, 2000). Scores range between 0 and 24. ASRS scores were available for 412 participants. The short version of the screening scale used in this study has good predictive validity for ADHD diagnosis and is considered a better tool for screening ADHD than the full version (Kessler et al., 2005, 2007).

#### 2.3.1 Neuropsychological measures

The participants were administered a neuropsychological test battery in one session lasting about three hours including one break. The tests were administered by psychologists or psychology students blind to information regarding perinatal risks or ADHD status and were trained by qualified neuropsychologists. The tests were administered in a fixed order. Most of the tests were pen-and-paper tasks. Attention measures also included tasks on a laptop. All tests were administered as instructed in the manuals. In WAIS-IV Vocabulary subtest every other question was used due to restricted time, and a total score was calculated by duplicating the score. In the Nine-Hole Peg Test the participants were to pick pegs from a container and place them in nine holes on a board as quickly as possible. Neuropsychological tests and outcome measures are presented in Table 1.

**Table 1:** Neuropsychological tests and outcome measures.

Cognitive domain	Test	Outcome measures	Function	Reference
Verbal reasoning	WAIS-IV	Similarities total score	Verbal reasoning	Wechsler (2008)
	WAIS-IV	Vocabulary total score	Verbal reasoning	Wechsler (2008)
	WAIS-IV	Information	Verbal reasoning	
Perceptual skills	WAIS-IV	Block Design	Perceptual reasoning	Wechsler (2008)
	WAIS-IV	Matrix Reasoning	Perceptual reasoning	
	ROCF	Copy score	Perceptual skills	Rey and Osterrieth, in Corwin and Bylsma (1993)
Memory	WMS-III	Logical Memory-I immediate and delayed recall score summed	Verbal memory	Wechsler (1997)
	WMS-III	Word List total and delayed score summed	Verbal memory	Wechsler (1997)
	ROCF	Immediate and delayed accuracy score summed	Visual memory	Rey (1941)
Working Memory	WAIS-IV	Digit Span Forward score	Short term verbal memory	Wechsler (2008)
	WAIS-IV	Digit Span Backward score	Verbal working memory	Wechsler (2008)
	WAIS-IV	Digit Span Sequencing score	Verbal working memory	Wechsler (2008)
Attention	CPT	Omission errors	Sustained attention/ vigilance	Mueller (2014) and Mueller and Piper (2014)
	CPT	Reaction time variability	Sustained attention/ vigilance	Mueller (2014) and Mueller and Piper (2014)
	Flanker Test	Flanker conflict cost: difference in reaction time between congruent and incongruent conditions.	Selective attention	Mueller and Piper (2014) and Mueller and Piper (2014)
Executive function	CPT	Commission errors	Inhibition of prepotent response	Mueller and Piper (2014) and Mueller and Piper (2014)
	Stroop	Completion time in the interference condition subtracted by completion time in the color naming condition. The sheets had 5 items on 20 rows. The items were read by rows.	Inhibition: Interference control	Stroop (1935)
	Word Fluency	Summed total number of generated animal words and words starting with the letter “K”	Cognitive flexibility: verbal fluency	Lezak et al. (2012)
Motor and Processing Speed	CPT	Reaction time average over correct responses	Motor speed	Mueller and Piper (2014) and Mueller and Piper (2014)
	WAIS-IV Coding	Total score	Processing speed	Wechsler (2008)
	Nine-Hole Peg Test	Best performance (time in seconds) out of four trials, two trials with each hand	Motor speed and coordination	Cutter et al. (1999)

WAIS-IV, Wechsler Adult Intelligence Scale 4th Edition; WMS-III, Wechsler Memory Scale 3rd Edition; ROCF, Rey–Osterrieth Complex Figure Test; CPT, Continuous Performance Task.

Two attention and EF tasks were given on a laptop using the PEBL test battery: Continuous Performance Task (CPT) and Flanker Test (Mueller, 2014; Mueller and Piper, 2014). In the CPT, participant sees individual letters displayed in the middle of the screen for varying durations and is instructed to press the space bar in response to all letters except the letter X. The CPT lasted for 7 min and had a target probability of 10%. In Flanker, horizontal arrows are shown on the screen and the participant is instructed to press the left arrow key if the arrow displayed in the middle of the screen points to the left, and right arrow key if the arrow points to the right. The task includes congruent and incongruent conditions, in which arrows on both sides of the target arrow point to the same or opposite direction.

The tasks comprised 21 neuropsychological outcome measures that formed seven domains: verbal reasoning, perceptual skills, memory, working memory, attention, executive function, and speed. Each domain consisted of three measures. Tests and outcome measures are listed in Table 1. The domains and measures were chosen based on literature (e.g., Biederman et al, 2011; Mostert et al., 2015; Fuermaier et al., 2018; LeRoy et al., 2019) and clinical experience of the authors. In addition to analyzing group means, we assessed cognitive deficits by identifying performance below 10th percentile or 1.5 SD of the control group performance (Doyle et al., 2000; Biederman et al., 2004), depending on the distribution of the outcome variable. A domain was considered deficient if two out of three measures were below the threshold.

### 2.4 Statistical analysis

IBM SPSS version 25 was used for all statistical analyses. Missing values on outcome measures were imputed with the mean of the corresponding childhood group. Logarithmic transformations were applied for variables that were non-normally distributed. Demographic variables were analyzed with chi-square tests or ANOVAs. Adjusted residuals were analyzed for *post hoc* comparisons in contingency tables and Bonferroni corrections applied for multiple comparisons (García-Pérez and Núñez-Antón, 2003). Multivariate analyses of variance (MANOVA) were conducted separately for each neuropsychological domain with the neuropsychological variables of that domain as outcome measures and childhood group status as the independent variable. These analyses were followed by ANOVAs for the neuropsychological outcome measures in each domain, if the MANOVA was significant. The Benjamini-Hochberg method was used to minimize type I error, with false discovery rate set at 0.05 (Benjamini and Hochberg, 1995). For effect sizes, Cohen’s *d* was used for continuous variables and Cramer’s *V* for contingency tables.

## 3 Results

A total of 481 participants were either lost to follow up or did not participate in the neuropsychological assessment. Attrition differed in childhood groups (χ^2^ = 14.213, *df* = 3, *p* < 0.003, *V* = 0.12). More individuals from the cAP group (*p* = 0.003) and fewer individuals from the Non-cAP group (*p* = 0.004) participated in the assessments than expected. There were more females in the study group (50.9%) than in those who did not participate (42.0%; χ^2^ = 7.35, *df* = 1, *p* = 0.007, *V* = 0.09).

The study groups did not differ significantly in sex, as shown in Table 2. The groups differed in age (Table 2), but as the age range is narrow (39 to 45 years), the finding was not considered meaningful in this age group. The groups did not differ in the number of birth risks (Table 2). Childhood socioeconomic status (SES) differed across the study groups (Table 2). In *post hoc* analyses fewer controls had the lowest SES status and more controls had the highest SES status than expected (*p* < 0.001 for SES class 1 and 3). Educational level differed across the groups (Table 2). In *post hoc* analyses significant differences were found in the lowest educational level with more individuals from the cADHD group (*p* < 0.001) and fewer individuals from the Non-cAP group (*p* = 0.004) than expected. The cADHD group reported more current ADHD symptoms than the other groups (Table 2).

**Table 2:** Demographical variables and current ADHD symptoms.

Variable	ADHD (1) *n* = 39		cAP (2) *n* = 79		Non-cAP (3) *n* = 255		Control (4) *n* = 69				Pairwise comparison^b^
	*M/n*	*SD/*%	*M/n*	*SD/*%	*M/n*	*SD/*%	*M/n*	*SD/*%	*p*	d*/V*	
Sex (female)	14	35.9	35	44.3	137	53.7	39	56.5	0.087	0.12	
Age	42.1	1.24	42.0	1.20	42.3	1.32	41.5	1.29	0.001	0.40	3 > 4
Number of birth risks											
1	17	43.6	55	69.6	161	63.4			0.09	0.11	
1	14	35.9	17	21.5	64	25.2					
3–5	8	20.5	7	8.9	29	11.4					
Childhood SES									0.002	0.15	
Childhood SES class 1	5	12.8	18	22.8	59	23.1	29	42.0			
Childhood SES class 2	12	30.8	16	20.3	74	29.0	21	30.4			
Childhood SES class 3	22	56.4	45	57.0	122	47.8	19	27.5			
Education									<0.001	0.25	
Basic	15	38.5	8	10.1	14	5.5	2	2.9			
Secondary	16	41.0	44	55.7	141	55.3	32	46.4			
Tertiary	8	20.5	27	34.2	100	39.2	35	50.7			
ASRS^a^	9.62	5.37	6.47	4.43	6.29	4.18	6.68	3.49	<0.001	0.42	1 > 2,3,4

cADHD, childhood ADHD; cAP, childhood attention problem; Non-cAP, no childhood ADHD or attention problems; SES, socioeconomic status; ^a^ASRS, Adult ADHD Self-Report Screening Scale; ADHD *n* = 33, cAP *n* = 72, Non-AP *n* = 243, Control *n* = 64. ^b^Pairwise comparison significant at *p* < 0.05.

In MANOVAs and subsequent ANOVAs, there was a significant overall group difference on all seven cognitive domains, with effect size estimates ranging from 0.27 to 0.62. (Table 3). Pairwise comparisons in 21 individual tests (corrected for multiple comparisons) are presented in Table 3. With 126 pairwise comparisons, the Benjamini-Hochberg-corrected *p* value criterion was 0.0142.

**Table 3:** Neuropsychological outcome measures.

Domain	Outcome measure	cADHD (1) *n* = 39	cAP (2) *n* = 79	Non-cAP (3) *n* = 255	Control (4) *n* = 69				Pairwise comparison^a^
		M (SD)	M (SD)	M (SD)	M (SD)	*F* (df = 3)	*p*	*d*	
Verbal reasoning						9.42	< 0.001	0.51	
	SM	27.44 (4.04)	28.09 (3.76)	29.15 (3.09)	30.22 (3.09)	8.19	< 0.001	0.47	1 < 3,4; 2 < 3,4
	VC	14.44 (4.10)	16.14 (4.26)	17.06 (3.74)	17.60 (3.28)	7.24	< 0.001	0.44	1 < 3,4
	IN	13.92 (4.88)	15.72 (4.47)	16.83 (4.33)	17.04 (4.14)	6.11	< 0.001	0.41	1 < 3,4
Perceptual skills						10.59	< 0.001	0.54	
	BD	43.38 (10.85)	45.41 (10.20)	48.60 (9.63)	51.84 (8.63)	8.75	<0.001	0.49	1 < 3,4; 2 < 3,4; 3 < 4
	MR	17.62 (4.64)	19.53 (3.93)	19.99 (3.89)	21.13 (2.80)	7.31	<0.001	0.44	1 < 2,3,4; 2 < 4
	ROCFc	32.96 (2.50)	33.18 (2.54)	33.66 (2.74)	33.76 (2.70)	1.38	0.26	0.19	
Memory						11.49	<0.001	0.56	
	LM	24.36 (9.48)	27.32 (7.35)	28.10 (7.09)	29.26 (7.60)	3.90	0.009	0.33	1 < 2,3,4
	WL	29.62 (6.16)	33.16 (5.36)	34.71 (5.31)	34.78 (6.08)	10.99	< 0.001	0.55	1 < 2,3,4
	ROCF	38.88 (10.93)	42.43 (12.29)	42.94 (12.32)	45.96 (13.60)	2.80	0.040	0.28	1 < 4
Working memory						5.19	0.002	0.38	
	DSF	9.21 (2.07)	9.38 (1.78)	9.61 (2.07)	9.70 (2.03)	0.70	0.55	0.14	
	DSB	8.95 (2.34)	9.08 (2.10)	9.48 (2.23)	9.97 (2.79)	2.40	0.07	0.26	
	DSS	8.0 (1.82)	8.96 (2.24)	9.36 (2.06)	9.32 (2.13)	5.11	0.002	0.38	1 < 3,4
Attention						3.39	0.018	0.31	
	CPTo	1.05 (1.36)	0.77 (1.52)	0.71 (1.17)	0.49 (0.83)	1.82	0.14	0.22	
	CPT RTV	97.82 (31.27)	85.59 (28.04)	85.97 (29.53)	87.72 (31.88)	2.33	0.074	0.26	
	FC	33.84 (64.30)	29.83 (55.20)	39.17 (51.79)	18.68 (58.80)	2.71	0.044	0.27	3 > 4
EF						5.75	0.001	0.40	
	CPTc	6.87 (3.17)	5.76 (2.92)	6.25 (3.32)	6.01 (2.98)	1.17	0.32	0.18	
	Stroop	54.28 (27.37)	43.22 (26.97)	42.88 (20.69)	41.36 (15.93)	3.41	0.018	0.31	1 > 2,3,4
	Word fluency	42.23 (10.83)	43.87 (9.81)	46.00 (9.81)	48.54 (9.29)	4.51	0.004	0.35	1 < 4; 2 < 4
Speed						13.85	<0.001	0.62	
	CPT RT	412.90 (55.92)	397.57 (47.02)	397.17 (45.29)	398.51 (46.49)	1.38	0.25	0.19	
	CD	63.26 (11.69)	68.94 (11.73)	73.04 (13.01)	77.55 (11.85)	13.04	<0.001	0.60	1 < 3,4; 2 < 3,4; 3 < 4
	9-HPT	12.13 (1.70)	11.67 (1.29)	11.49 (1.20)	11.55 (1.27)	3.93	0.009	0.33	1 > 3,4

cADHD, childhood ADHD; cAP, childhood attention problem; Non-cAP, no childhood ADHD or attention problems; SM, similarities, VC, vocabulary, IN, information, BD, Block Design, MR, Matrix Reasoning, ROCFc, Rey–Osterrieth Complex Figure Test Copy, LM, Logical Memory, WL, Word List Learning, ROCF, Rey–Osterrieth Complex Figure Test, DSF, Digit Span Forward, DSB, Digit Span Backward, DSS, Digit Span Sequencing, CPTo, Continuous Performance Task Omission Errors, CPT RTV, Continuous Performance Task Reaction Time Variability, FC, Flanker Test Conflict Cost, CPTc, Continuous Performance Task Commission Errors, CPT RT, Continuous Performance Task Reaction Time, CD, Coding, 9-HPT, Nine-Hole Peg Test. ^a^Pairwise comparisons significant at *p* < 0.0142 after controlling for multiple comparisons.

The cADHD group performed poorer than the control group and the Non-cAP group in 11 out of 21 test measures including 5 reasoning and two long-term memory measures, one working memory measure, one executive functions measure, and two measures assessing psychomotor and processing speed (Table 3). In addition, the cADHD group performed poorer than the control group in one other working memory and one executive functions measure. The cAP group obtained better scores than the cADHD group in four test measures: one perceptual reasoning measure, two long-term memory measures, and one executive functions measure. The cAP group performed poorer than the Non-cAP and control groups in three measures: one verbal and one perceptual reasoning measure, and in one processing speed measure. In addition, the cAP performed poorer than the control group in one other perceptual reasoning measure and one executive functions measure. The Non-cAP group obtained lower scores than the control group in three measures: one perceptual reasoning, one attention, and one processing speed measure.

The number of deficient cognitive domains in a single participant ranged from zero to six (out of seven). Deficient domains were stratified to 0, 1, 2, and 3 or more deficient domains (Table 4). The groups differed in the number of deficient domains (χ^2^ = 40.56, *df* = 9, *p* < 0.001, *V* = 0.18). There were more participants than expected with zero deficits in the Non-cAP group and fewer than expected participants with zero deficits in the cADHD group. There were more participants than expected with 3 or more deficits in the cADHD group. The ASRS score correlated with the total number of deficient cognitive domains (*r* = 0.19, *p* < 0.001).

**Table 4:** Total number of deficient cognitive domains in childhood groups.

Number of deficient cognitive domains	ADHD (1)		cAP (2)		Non-cAP (3)		Control (4)	
	*n*	%	*n*	%	*n*	%	*n*	%
0	13	33.3*	44	55.7	185	72.5*	52	75.4
1	11	28.2	21	26.6	40	15.7	10	14.5
2	6	15.4	9	11.4	20	7.8	3	4.3
3 or more	9	23.1*	5	6.3	10	3.9	4	5.8

cADHD, childhood ADHD; cAP, childhood attention problem; Non-cAP, no childhood ADHD or attention problems. *Post hoc analysis significant at *p* < 0.004.

**Figure 2:**
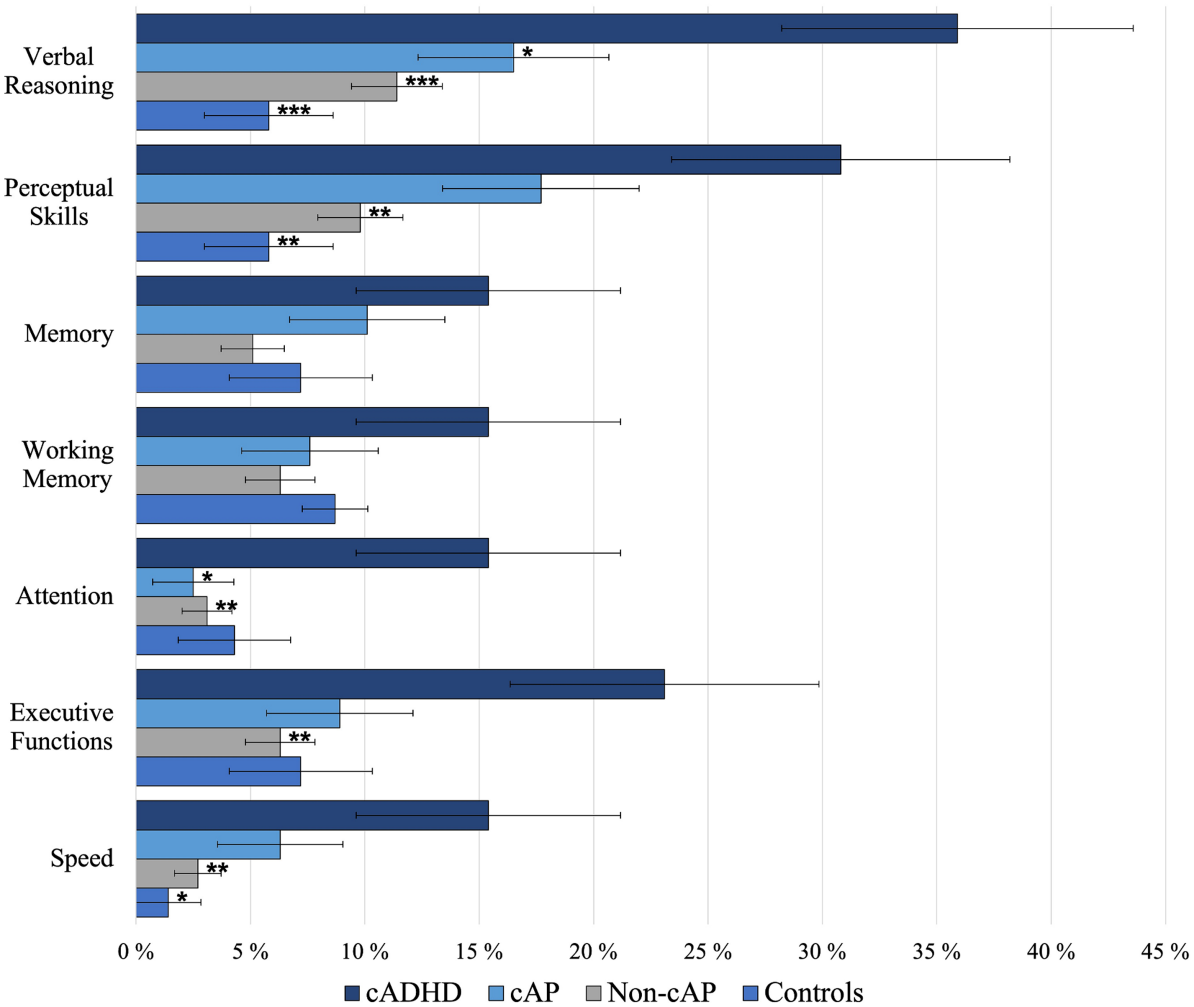
Mean percentage of deficient cognitive domains. Pairwise comparisons are shown for the ADHD group compared to other groups. *p < 0.05; **p < 0.01; ***p < 0.001. Error bars show standard errors.

The percentage of participants with deficient functioning (two out of three measures deficient within a domain) differed by childhood group in the following domains: verbal reasoning (χ^2^ = 21.74, *p* < 0.001, *V* = 0.22), perceptive skills (χ^2^ = 18.47, *p* < 0.001, *V* = 0.20), attention (χ^2^ = 13.09, *p* < 0.004, *V* = 0.17), executive functions (χ^2^ = 12.60, *p* < 0.006, *V* = 0.17), and speed (χ^2^ = 15.30, *p* < 0.002, *V* = 0.19). There were no group differences in the domains memory (χ^2^ = 6.56, *p* < 0.09, *V* = 0.12) and working memory (χ^2^ = 4.07, *p* < 0.25, *V* = 0.10). In pairwise comparisons, only the cADHD group differed significantly from the other groups with greater number of individuals having deficits than the other groups in five out of seven domains (Figure 2). The cAP group had fewer deficits than the cADHD group in domains verbal reasoning and attention. The Non-cAP group had fewer deficits than the cADHD group in domains verbal reasoning, perceptual skills, attention, executive functions, and speed, and the control group had fewer deficits than the cADHD group in domains verbal reasoning, perceptual skills, and speed (Figure 2).

## 4 Discussion

This longitudinal study investigated neuropsychological functioning of 40-year-old adults with childhood ADHD or subthreshold symptoms and perinatal risks. Comparisons to cohort members with similar perinatal risks in childhood and to controls without perinatal risks revealed lower cognitive performance for the ADHD group. This difference was present in a broad array of cognitive domains and displayed as cognitive deficits. Differences with the largest effect sizes were found in verbal reasoning, perceptual skills, memory, and processing speed. The subthreshold ADHD group had fewer cognitive deficits than the ADHD group but more than the controls.

Based on our results, childhood ADHD is associated with cognitive dysfunction in mid-life. Overall, there were clear differences in average scores and numbers of cognitive deficits between the cADHD and the Non-cAP and control groups. The cADHD group performed poorer than the Non-cAP and control groups in over half of all neuropsychological test measures. The majority of the cADHD group had at least one cognitive deficit, whereas the majority of the Non-cAP group displayed no deficits. Similarly to our findings, previous longitudinal studies have shown individuals with childhood ADHD to score lower in measures of intelligence, executive functions and working memory (Barkley et al., 2008; Biederman et al., 2012; Miller et al., 2012; Moffitt et al., 2015). These consistent findings from studies following children with ADHD into adulthood underline the need to manage cognitive dysfunction and the impairment associated with it early on. Clinicians are more informed in assessing and treating adults with childhood ADHD knowing the long-term cognitive burden associated with childhood ADHD.

In general, the performance of the subthreshold ADHD group fell between the ADHD and control groups. The cAP group differed from both the cADHD and the Non-cAP and control groups in only a few measures, did not show manifest clearly differing proportions of cognitive deficits, and had average scores often between those obtained by the cADHD and Non-cAP groups. The subthreshold group performed poorer than controls in one verbal and two visual reasoning measures, verbal fluency, and processing speed. Previous studies have investigated the association of cognitive performance with current subthreshold ADHD symptoms in childhood or adulthood, not current cognitive functioning to previous ADHD symptoms like this study. Comparing our findings to these studies reveal similar patterns of cognitive functioning. In adults, few group differences in neuropsychological performance were found between individuals with subthreshold ADHD and those with the full syndrome (Faraone et al., 2006). In children, subthreshold ADHD has been linked to poorer performance compared to controls in processing speed and verbal and spatial problem solving (Biederman et al., 2018; Kirova et al., 2019). Our finding of poorer processing speed is in line with a study suggesting basic information processing skills to be worse with increasing ADHD symptoms in children and adolescents (Salum et al., 2014). A novel finding was lower performance in verbal fluency, implying deficient functioning in one area of executive functioning for the subthreshold group. However, a study focusing on current subthreshold ADHD symptoms in adults did not indicate poor executive functions (Schneidt et al., 2020). Examination of deficient cognitive functioning revealed the subthreshold group to have fewer individuals with no deficient cognitive domains compared to the remaining cohort and the control group. For an individual, childhood subthreshold ADHD symptoms might thus be a risk factor for reduced cognitive functioning later in life. However, the level of cognitive functioning does not appear to be as low as in the ADHD group, as has also been suggested in the childhood literature (Balázs and Keresztény, 2014). Future studies should continue to investigate long-term cognitive functioning in subthreshold childhood ADHD and functional impairment associated with it.

Perinatal risks were not generally associated with poorer overall cognitive functioning as the group with no or low levels of childhood ADHD symptoms performed similarly to the control group. The Non-cAP and control groups obtained similar results in average test scores and deficit proportions differing in only three test measures. There were few areas with lower performance compared to the control group, namely spatial reasoning, selective attention, and processing speed. Perinatal risks, such as low birth weight, preterm birth, and low Apgar score have been associated with impaired cognitive performance in adulthood (Ehrenstein et al., 2009; Allin et al., 2011; Pyhälä et al., 2011). The risk cohort in this study had more problems at school than controls, with several early risk factors increasing the likelihood of school problems (Lindahl and Michelsson, 1987). Our perinatal risk group varies in etiology and future studies should address the role of different and multiple early risk factors on adult cognition. Nevertheless, our findings hint at processing speed, complex attention and visuospatial problem solving being affected in these adults.

In the domains verbal reasoning and perceptual skills, the ADHD group had poorer mean performance than controls on all reasoning measures. Furthermore, over 30% of the group displayed a deficit in verbal reasoning or perceptual skills, when defined as performance below 10th percentile or 1.5 SD of the control group. This represented the largest deficit proportions over all cognitive domains. Lower reasoning, or intelligence, reflecting lower cognitive abilities in the ADHD group, have been established in meta-analyses (Frazier et al., 2004; Hervey et al., 2004; Bridgett and Walker, 2006; Pievsky and McGrath, 2018). Poorer reasoning abilities could reflect a global cognitive deficit or various specific cognitive problems resulting in poor performance on measures requiring complex cognitive skills. In one meta-analysis, individuals with childhood-diagnosed ADHD performed more poorly than those without confirmed childhood ADHD (Bridgett and Walker, 2006). Our finding is in line with this result, indicating that established childhood ADHD symptoms are associated with lower reasoning abilities in adulthood.

The groups differed in the memory domain that included verbal and visual tasks. This finding is in line with previous studies of ADHD adults demonstrating medium effect size for deficits in verbal memory and small effect size for visual memory (Hervey et al., 2004; Schoechlin and Engel, 2005). Especially verbal learning, measured with word list learning, was affected in the ADHD group with poorer performance compared to all other groups and a medium effect size. Learning a set of unrelated words requires focused and sustained attention, executive functions, and different cognitive memory components (Pollak et al., 2007; Skodzik et al., 2017). Impaired performance on this measure is thus consistent with a notion of neuropsychological performance declining with increasing cognitive demands in adults with ADHD (Hervey et al., 2004). Long-term memory problems in adult ADHD appear to be related to encoding and learning (Fuermaier et al., 2013; Kim et al., 2014; Skodzik et al., 2017). In children with ADHD, poor performance in word list learning has been linked to lack of effortful learning strategies (Egeland et al., 2010). In adults with ADHD, strategic memory retrieval processes, which rely on executive functions, have been found to be deficient (Pollak et al., 2007). Together, our results and those outlined above suggest that cognitive complexity and high demand on executive functions may underlie poor memory performance in adults with ADHD. Interestingly, only 15% of the ADHD group in this study had deficits in the memory domain, whereas in the domains verbal reasoning (36%) and perceptual skills (31%) the proportions were twofold. This could simply imply that memory problems are not as common as problems in complex reasoning. The likeliest explanation, however, is that the higher number of individuals with deficits in verbal and visual measures reflects the complexity of these measures and the high demands on multiple cognitive domains (Friedman et al., 2006; Buczyłowska et al., 2020).

A similar explanation of increased complexity of tasks causing deficient performance arises for working memory. The groups differed in working memory, with the Digit Span Sequencing the only measure where pairwise comparisons resulted in poorer mean performance for the ADHD group compared to controls. Working memory performance has been impaired in ADHD adults also in other follow-up studies (Barkley et al., 2008; Miller et al., 2012; Moffitt et al., 2015). Simple short-term memory performance was similar in all groups and group differences only appeared in the Digit Span tasks requiring mental manipulation. Our results are consistent with the idea of executive function demands increasing group differences in working memory performance in ADHD (Alderson et al., 2013). Our findings suggest that childhood ADHD is associated with lower working memory performance in complex tasks in adulthood.

Executive functions differed between the groups both in group means and in frequencies of cognitive deficits. Impairment in executive functions is a consistent finding in the ADHD literature (Willcutt et al., 2005; Adler et al., 2017; Pievsky and McGrath, 2018). The ADHD group performed poorer than all other groups in interference control and poorer than the control group in verbal fluency, but the effect sizes were in the small range. The small effect sizes in group-level analysis might reflect variability in different executive function tasks within ADHD, a reoccurring finding in studies of neuropsychological performance in ADHD (Boonstra et al., 2010; Mostert et al., 2015). The poor performance in interference control is in line with a previous study indicating that response inhibition difficulties persist from childhood to adolescence regardless of ADHD symptom development (McAuley et al., 2014). Although effect sizes for executive functions were small, 23% of the ADHD group displayed deficient functioning in the tasks. This result is similar to deficit estimates in two other studies reporting 24–31% impairment in executive functions for adults with ADHD (Biederman et al., 2006; Halleland et al., 2019). Together, our results suggest that when studied as a group or through single test measures, individuals with ADHD might not differ from controls due to high variation in performance. A similar pattern emerges for the attention measures: the average performance of the groups did not differ, but a higher number of participants in the ADHD group had deficits within this domain. The relatively high proportion of individuals showing deficits in executive functioning and attention implies that these deficits are likely to be impairing for daily functioning.

Another domain with lower performance for the ADHD group compared to controls was processing speed. WAIS-IV Coding task had the highest effect size out of all measures. The Coding task requires focused and sustained attention and visual short-term memory, and poor performance on it has been found in adults with ADHD (Theiling and Petermann, 2016; Anker et al., 2022). Again, only 15% of the ADHD group had deficient scores in the speed domain compared to 31% in the perceptual skills domain suggesting a similar pattern of complex tasks causing more deficits as discussed above. Nevertheless, deficits in basic cognitive processes in ADHD, especially processing speed, might underlie deficits in higher order processes needed in, for example, WAIS-IV Block Design, a task used in the perceptual skills domain in this study (Mulder et al., 2010; Butzbach et al., 2019; Mohamed et al., 2021). Our finding of slower processing speed for ADHD is consistent with Hervey et al.’s (2004) hypothesis of task components important in determining the difficulty of a task for adults with ADHD (complexity, time requirements, processing speed, and a motor component). This hypothesis is supported by poor performance but smaller effect size on the Nine-Hole Peg Test, which has a motor component and a time constraint but is less complex than the Coding task. Processing speed tasks have shown promise for screening ADHD adults in psychiatric populations and for testing the effects of ADHD medication (Wiig and Nielsen, 2012; Nielsen et al., 2017). Future studies should address whether this is a sensitive task for age-related slowing in ADHD adults. Two test measures used in this study differentiated the two ADHD symptom groups from non-symptom groups and all cohort groups from the control group. Future studies could explore the potential of visuospatial reasoning visual processing speed tasks as useful screening measures for cognitive difficulties in adulthood.

One-fourth of the ADHD group had deficits in three or more cognitive domains and over half had at least one deficit. Our results are in line with evidence of individuals with ADHD exhibiting high numbers of cognitive deficits when performance is examined on a wide-ranging test battery (Nigg et al., 2005b; Fuermaier et al., 2015) and suggest this also generalizes to adults with childhood-established ADHD. Most previous research on adults has focused on investigating individual tests or studied certain cognitive domains, mostly executive functions and attention (Fischer et al., 2005; McAuley et al., 2014). Cognitive deficits pose a risk for low socioeconomic status and poor academic and occupational functioning, long-term outcomes often associated with ADHD (Shaw et al., 2012; Uchida et al., 2018). This seems to be the case also in our cohort, as we have previously reported that the ADHD group had lower grade average in comprehensive school compared to the other groups (Schiavone et al., 2019). It is noteworthy, however, that one-third of the ADHD group did not show deficits in any of the domains. This is expected because of the complexity and heterogeneity of cognitive functioning in ADHD: certain individuals appear to be severely impaired whereas others perform similarly to non-affected peers (Coghill et al., 2014). A potential area for future research is to investigate deficit profiles, which we were unable to explore within the scope of this study. It would be useful for clinicians to have information on possible deficit patterns and the likelihood of certain cognitive deficits coexisting with others. Nonetheless, our results highlight the importance of examining deficient performance in addition to group-level differences to better understand the clinical and functional implications of low cognitive performance associated with ADHD.

Current self-reported ADHD symptoms correlated with the number of cognitive deficits. The childhood ADHD group reported more ADHD symptoms in adulthood and in a separate study also reported more problems in executive functioning than the remaining cohort (Schiavone et al., 2019). This correlation is not always present and previous studies have suggested that cognitive performance does not depend on or predict how ADHD symptoms appear over time, and that cognitive performance is trait-like, as are ADHD symptoms (Biederman et al., 2009; Van Lieshout et al., 2013; McAuley et al., 2014; Uchida et al., 2018). In line with this view, the group with childhood subthreshold ADHD in our study did not report elevated ADHD symptoms in adulthood, but still presented subtle cognitive dysfunction. The correlation between cognitive functioning and concurrent ADHD symptoms might also be due to questions in the ADHD screener scale tapping into other psychiatric disorders, such as depression or anxiety, which can also be linked to lower cognitive functioning (Bridgett and Walker, 2006). Also, in our cohort, elevated levels of depression, anxiety and stress were previously found to be linked to higher ADHD symptoms (Schiavone et al., 2019). Future studies could explore how self-reported ADHD and other psychiatric symptoms and their association with cognitive performance develop over time in individuals with high or subthreshold levels of ADHD symptoms.

### 4.1 Strengths and limitations

This study has some potential limitations. The ADHD diagnosis relied on information gathered in the context of the research project but was done retrospectively. This was because ADHD was not established as a disorder in its current form during the childhood follow-ups. Childhood socioeconomic status was lower in our risk cohort compared to controls, which might affect the results. However, no differences were found across the groups with or without ADHD symptoms and they can be considered similar in childhood family and social background. More individuals from the cohort with no attention problems and fewer individuals with subthreshold problems took part in the neuropsychological assessment, which might have skewed the results. Participating required time and effort and may have discouraged those with more challenges to take part. Had more cohort members from the ADHD and subthreshold groups participated, group differences might have been even clearer. More females participated in the neuropsychological assessment than were lost to follow up, creating another possible bias in the results. However, there were no significant gender differences across the study groups. The cADHD group was less educated than the remaining cohort, which is a common finding in ADHD studies (Barkley et al., 2008; Bussing et al., 2010). Low education may impair results in cognitive tests, but it is also likely that cognitive difficulties apparent in school age have affected educational attainment. The latter conclusion is supported by an earlier study of a sample of the risk cohort that reported more need for extra help at school at the age of nine, and a recent study of the childhood ADHD groups that reported lower grade average after comprehensive school (Michelsson and Lindahl, 1987; Schiavone et al., 2019). We did not assess possible psychiatric disorders apart from the most severe as individuals with psychotic disorders were excluded from the study. ADHD in general and persistent ADHD in adulthood have been linked to a higher risk of psychiatric comorbidities (Yoshimasu et al., 2018; Kooij et al., 2019). However, we reported no differences between the groups on a subjective measure of depression, anxiety, and stress in our previous study (Schiavone et al., 2019). The cohort in this study consisted of individuals with perinatal risks and generalizations to other populations should be made with caution.

The strengths of this study include the longitudinal setting and comparisons to groups with similar demographical and perinatal risk background with and without ADHD symptoms. The length of the follow-up is a significant strength of the study as longitudinal studies on ADHD extending beyond young adulthood are rare. Another strength is the comprehensiveness of the neuropsychological test battery which included domains that are less studied in adult ADHD. A broad neuropsychological test battery is more likely to gauge the variation and complexity of neuropsychological functioning in ADHD (Willcutt et al., 2005; Sonuga-Barke and Coghill, 2014).

### 4.2 Conclusion

To conclude, among subjects with perinatal risks, childhood ADHD was linked to deficits and poorer performance in various cognitive domains in 40-year-old adults compared to healthy controls. Over half of adults with childhood ADHD showed neuropsychological dysfunction in at least one cognitive domain. Tasks requiring complex cognitive skills resulted in more deficient functioning within the ADHD group. Childhood subthreshold ADHD was associated with fewer deficits than childhood ADHD, but more deficits compared to controls. This study adds to the limited knowledge of lifespan neuropsychological functioning in childhood ADHD and subthreshold symptoms.

## Data availability statement

The datasets presented in this article are not readily available due to the possibility of individuals being identified by combining health-related data. Requests to access the datasets should be directed to nella.schiavone@helsinki.fi.

## Ethics statement

The studies involving humans were approved by Ethical Review Board of the Helsinki and Uusimaa hospital district (number 147 /13/03/00/13). The studies were conducted in accordance with the local legislation and institutional requirements. The participants provided their written informed consent to participate in this study.

## Author contributions

NS: Writing – review & editing, Writing – original draft, Methodology, Conceptualization. MV: Writing – review & editing, Supervision. SL: Writing – review & editing, Methodology, Conceptualization. JL: Writing – review & editing, Data curation, Conceptualization. RV: Writing – review & editing, Methodology. AT-H: Writing – review & editing. IJ: Writing – review & editing, Investigation. EL: Writing – review & editing, Investigation. LH: Writing – review & editing, Supervision, Project administration, Methodology, Investigation, Funding acquisition, Data curation, Conceptualization.
